# Emergency Laparoscopic Cholecystectomy for Acute Cholecystitis with Hepatic Segment 4 Hypoplasia and Gallbladder-Attached Accessory Liver: A Rare Dual Anomaly Case

**DOI:** 10.70352/scrj.cr.26-0001

**Published:** 2026-05-12

**Authors:** Koki Miya, Takeshi Kato, Fumitaka Yagi, Takashi Saitoh, Katsuhiko Suzuki

**Affiliations:** Department of General Surgery, Honjo Daiichi Hospital, Yurihonjo, Akita, Japan

**Keywords:** hepatic hypoplasia, accessory liver, acute cholecystitis, laparoscopic surgery, case report

## Abstract

**INTRODUCTION:**

Hepatic hypoplasia and accessory liver tissue are rare congenital anomalies, and their coexistence with acute cholecystitis is exceedingly uncommon. We report a case of acute cholecystitis in a patient with segment 4 hypoplasia and an accessory liver attached to the gallbladder serosa in whom laparoscopic cholecystectomy was performed safely.

**CASE PRESENTATION:**

A 76-year-old woman presented with acute cholecystitis. CT revealed marked hypoplasia of hepatic segment 4 accompanied by mild hypoplasia of the anterior hepatic segment. Because the intestinal tract was positioned anterior to the gallbladder, percutaneous transhepatic gallbladder drainage was considered difficult, and emergency laparoscopic cholecystectomy was undertaken. Intraoperatively, the gallbladder was displaced to the right, and the duodenum overlapped the gallbladder neck ventrally. These anatomic relationships initially limited visualization and raised concern regarding the need to convert to open surgery. However, adequate exposure was obtained by retracting the lateral hepatic segment to the left using a fan-shaped retractor, placing the patient in the reverse Trendelenburg position, and caudally retracting the duodenum with forceps. With these adjustments, laparoscopic cholecystectomy was completed safely. On the serosal surface of the gallbladder, a reddish-brown mass connected to the liver by a cord-like structure was identified and excised en bloc using energy devices. Histopathological examination confirmed the mass to be accessory liver tissue. The patient’s postoperative course was uneventful.

**CONCLUSIONS:**

Because accessory liver tissue may contain portal structures and can occasionally harbor malignancy, resection using energy devices is advisable. This case highlights the importance of careful preoperative imaging, meticulous intraoperative anatomical assessment, and appropriate technical modifications when managing patients with rare congenital hepatic anomalies. Surgeons should be aware that such anomalies can alter hepatobiliary anatomy, restrict therapeutic options, and increase operative complexity, necessitating adaptable operative strategies.

## INTRODUCTION

Hepatic hypoplasia and accessory liver tissue are rare congenital anomalies that are most often asymptomatic and discovered incidentally.^1,2)^ However, during hepatobiliary surgery, including cholecystectomy, these anomalies may significantly affect operative exposure and influence surgical technique. Although each of these anomalies has been reported individually, the intraoperative implications of their coexistence have not been sufficiently described. In particular, it remains unclear how simultaneous anatomical variations may impact surgical strategy and technical difficulty during laparoscopic cholecystectomy. Herein, we describe a case of acute cholecystitis in a patient with hypoplasia of hepatic segment 4 and an accessory liver attached to the gallbladder serosa in whom laparoscopic cholecystectomy was performed safely.

## CASE PRESENTATION

**Fig. 1:**
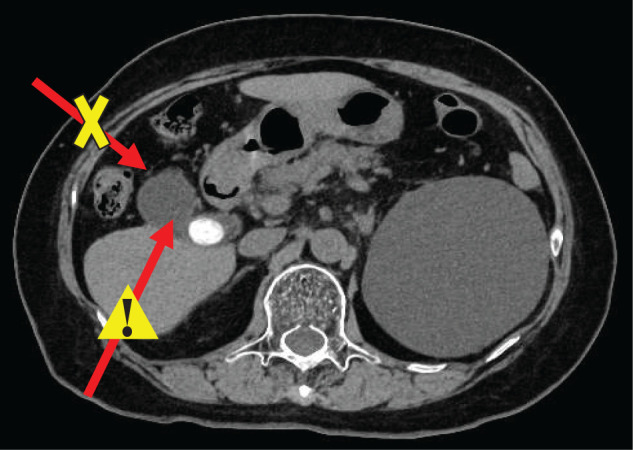
Abdominal CT findings. Abdominal CT showing hypoplasia of hepatic segment 4 and gallbladder distension with impacted gallstones. The arrows indicate the potential percutaneous puncture routes. The anterior approach (arrow with a cross) is not feasible due to bowel interposition. The posterior approach (arrow with a warning sign) is considered unsafe because of the risk of pneumothorax. These findings suggest that percutaneous gallbladder drainage is technically difficult in this case.

A 76-year-old woman presented with right upper-quadrant pain and vomiting for 4 days. On admission, her body temperature was 38.4°C. Physical examination revealed a flat and soft abdomen with epigastric tenderness. Laboratory tests revealed leukocytosis (13000/μL; normal 3300–9000/μL) and an elevated C-reactive protein level (18.9 mg/dL; normal <0.03 mg/dL). Abdominal CT demonstrated gallbladder distension with an impacted gallstone and wall thickening consistent with acute cholecystitis. Imaging also revealed marked hypoplasia of hepatic segment 4, accompanied by mild hypoplasia of the anterior hepatic segment. Because the intestinal tract was positioned anterior to the gallbladder, percutaneous transhepatic gallbladder drainage was considered difficult (**Fig. 1**). Because the duration of symptoms exceeded 72h, this case was classified as moderate (Grade II) acute cholecystitis according to the Tokyo Guidelines 2018, and early surgical intervention was selected.

**Fig. 2:**
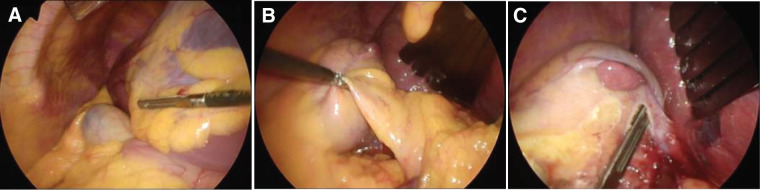
Operative field exposure. Intraoperative findings showing altered anatomical relationships due to hepatic segment 4 hypoplasia, including rightward deviation of the falciform ligament and gallbladder, with the duodenum overlying the gallbladder neck (**A**). Adequate visualization was achieved by retracting the lateral hepatic segment to the left using a fan-shaped retractor, placing the patient in the reverse Trendelenburg position, and retracting the duodenum caudally with laparoscopic forceps (**B**). A reddish-brown mass was identified on the serosal surface of the gallbladder (**C**). The mass was connected to the liver parenchyma via a cord-like structure and was safely dissected using an ultrasonic coagulation and dissection device.

An emergency laparoscopic cholecystectomy was performed. Intraoperatively, hypoplasia of segment 4 and the anterior segment resulted in rightward deviation of the falciform ligament and gallbladder. The duodenum overlapped the gallbladder neck ventrally (**Fig. 2A**). These anatomic variations limited visualization and raised concern regarding possible conversion to open surgery. To improve exposure, an additional port was placed, converting the procedure to a 5-trocar approach. The lateral hepatic segment was retracted to the left using a fan-shaped retractor, while the patient was placed in the reverse Trendelenburg position. The duodenum was retracted caudally with forceps (**Fig. 2B**). These maneuvers improved visualization sufficiently to obtain the critical view of safety, after which the cystic duct and artery were divided and the gallbladder was dissected from its bed using a standard laparoscopic cholecystectomy technique.

**Fig. 3:**
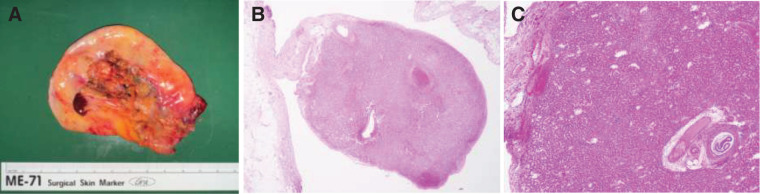
Features of the identified mass. A reddish-brown mass connected to the serosal surface of the gallbladder (**A**). Histopathological examination revealed that the mass was composed of normal hepatic tissue, consistent with an accessory liver mass (**B**, **C**; hematoxylin–eosin staining).

A reddish-brown mass was observed on the gallbladder serosa, connected to the liver parenchyma by a cord-like structure (**Fig. 2C**). This structure was divided using an ultrasonic coagulation and dissection device, and the mass was removed en bloc with energy devices. Histopathological examination revealed normal hepatic parenchyma, confirming the diagnosis of an accessory liver (**Fig. 3**). The patient’s postoperative course was uneventful. She was discharged on POD 7, and no complications were observed during 1 year of follow-up.

## DISCUSSION

Hepatic hypoplasia and an accessory liver are rare congenital anomalies. To the best of our knowledge, emergency laparoscopic cholecystectomy in the presence of both hepatic segment 4 hypoplasia and accessory liver has rarely been reported.

Hepatic lobar or segmental agenesis/hypoplasia has been reported in the literature with a low incidence of 0.005%.^1)^ Although generally asymptomatic, segment 4 hypoplasia may be associated with abnormal positioning of the gallbladder, which could potentially contribute to bile stasis and gallstone formation due to cystic duct compression or torsion.^3)^ However, only limited reports have described an association between hepatic segment 4 hypoplasia and cholelithiasis or acute cholecystitis, and the underlying mechanism remains unclear. In the present case, abnormal positioning of the gallbladder was observed, and its potential contribution to gallstone formation was considered; however, a definitive causal relationship could not be established.

A previous report has identified segment 4 hypoplasia as a risk factor for bile duct injury during laparoscopic cholecystectomy, emphasizing the importance of obtaining the critical view of safety before division.^1)^ Although the underlying mechanism has not been explicitly described, this increased risk is likely attributable to abnormal gallbladder positioning, which alters the conventional operative field. Therefore, in patients with hepatic developmental anomalies, securing adequate visualization is essential for safely performing laparoscopic cholecystectomy. In this case, segment 4 hypoplasia resulted in rightward deviation of the gallbladder, with the duodenum overlapping the gallbladder neck, making both percutaneous drainage and initial laparoscopic exposure difficult. However, adequate visualization was achieved by retracting the lateral hepatic segment to the left using a fan-shaped retractor, placing the patient in the reverse Trendelenburg position, and retracting the duodenum caudally with laparoscopic forceps. These maneuvers, facilitated by the use of 5 trocars, provided stable operative exposure without excessive repositioning. Although such cases are uncommon, if hepatic hypoplasia predisposes patients to gallstone formation, similar presentations may occur. Thus, insights gained from this case may be valuable in guiding future management.

An accessory liver was incidentally identified on the serosal surface of the gallbladder. Accessory liver occurs in approximately 0.7% of individuals^2)^ and is usually asymptomatic; however, it has occasionally been associated with hepatocellular carcinoma.^2,4,5)^ Therefore, incidental resection is recommended. Pathological studies have shown that accessory liver tissue may contain portal triads and maintain vascular and biliary connections with the main liver.^2,6)^ Accordingly, safe resection requires careful control of vascular and biliary structures using clips or energy devices.^6)^ In our case, the accessory liver was attached to the gallbladder and could be safely resected en bloc without technical difficulty. Transection was limited to the peripheral portion of the liver; therefore, the accessory liver was excised using an ultrasonic coagulation and dissection device to minimize the risk of bleeding and bile leakage, with careful attention to the control of potential vascular and biliary structures. No complications related to the accessory liver were observed; however, this case highlights the importance of careful intraoperative identification and safe resection of accessory liver tissue during cholecystectomy.

Importantly, although no direct causal relationship between hepatic segment 4 hypoplasia and accessory liver can be established, their coexistence in this case resulted in compounded intraoperative challenges. This case provides clinically relevant insight into the intraoperative impact of such coexisting anomalies. Segment 4 hypoplasia limited adequate exposure of the gallbladder neck due to abnormal anatomical positioning, while the accessory liver attached to the gallbladder introduced additional complexity requiring careful dissection and secure control of potential vascular and biliary structures. These independent factors collectively increased the technical difficulty of the procedure and necessitated modifications to the standard surgical approach to safely achieve the critical view of safety.

A limitation of this case is that contrast-enhanced CT was not performed due to the emergency setting, which limited detailed evaluation of vascular anatomy. Preoperative vascular assessment might have provided additional anatomical information.

## CONCLUSIONS

We presented a case of acute cholecystitis associated with 2 rare hepatic anomalies: segment 4 hypoplasia and an accessory liver. Laparoscopic cholecystectomy can be safely performed in such cases when preoperative imaging, intraoperative anatomical assessment, and targeted technical modifications are implemented. Although rare, these anomalies may be encountered unexpectedly, and ensuring safety requires flexible intraoperative adjustments. This case underscores the importance of recognizing unusual anatomical variations and adapting surgical strategies accordingly, thereby contributing to safer hepatobiliary practice.

## DECLARATIONS

### Availability of data and materials

All relevant data are included in the article. No additional data are available.
